# Safety of Patient-Controlled Analgesia After Surgery in Children And Adolescents: Concerns And Potential Solutions

**DOI:** 10.3389/fped.2018.00336

**Published:** 2018-11-06

**Authors:** Don Daniel Ocay, Annik Otis, Alisson R. Teles, Catherine E. Ferland

**Affiliations:** ^1^Department of Experimental Surgery, McGill University, Montreal, QC, Canada; ^2^Shriners Hospitals for Children-Canada, Montreal, QC, Canada; ^3^Department of Anesthesia, McGill University, Montreal, QC, Canada; ^4^Department of Anesthesia, Montreal Children's Hospital, Montreal, QC, Canada; ^5^Integrated Program in Neuroscience, McGill University, Montreal, QC, Canada; ^6^Child Health and Human Development, Research Institute-McGill University Health Centre, Montreal, QC, Canada

**Keywords:** patient-controlled analgesia, pediatrics, safety, surgery, concerns, solutions

## Abstract

Patient-controlled analgesia (PCA) is common practice for acute postoperative pain management. Postoperative PCA use decreases pain intensity and improves patient satisfaction when compared to non-PCA routes of medication administration. Although PCA has several advantages regarding efficacy and safety, adverse events remain a concern. Programming errors and protocols, patient monitoring, and PCA by proxy or with continuous infusion are recurring silent dangers of PCA use in children and adolescents. Innovative considerations need to be emphasized for future improvement of PCA devices for elective surgical patients. With technology within the healthcare setting advancing at a fast pace, smart pump technology is something to look forward to.

## Introduction

Patient-controlled analgesia (PCA) was clinically introduced to adults in 1971 to quickly and effectively relieve postsurgical pain. Today, PCA is common practice for managing acute pain of hospitalized children and adolescents in different contexts such as postoperative, oncology, trauma and palliative care (1). The PCA allows the patient to self-administer analgesics, usually intravenous opioids, by using a programmable computerized pump. Giving patients the control over analgesic drug administration allows for better titration to maximize pain relief and minimize risk of overdose (2). This active self-management of pain is an important factor concerning patients' psychological well-being since the patient can receive pain medication immediately without the need for a nurse to administer it. Use of PCA after surgery decreases pain intensity and improves patient satisfaction when compared to non-PCA routes of opioid administration (3).

Although PCA has several advantages in terms of efficacy and safety (4, 5), adverse events remain a concern (6). In a meta-analysis from the Cochrane database, it was demonstrated that the incidence of opioid-induced adverse events is similar in PCA compared to non-PCA, with the exception of pruritus, which was increased with PCA (3). Optimizing the safety of PCA use with children and adolescents without compromising its efficacy is still a challenge for researchers and developers (7). Selection and programming of the pump, monitoring and selection of patients, staff and patient/parent education, as well as the use of PCA by proxy are recurrent themes for safe PCA use. In this mini-review we will highlight safety issues of PCA use and suggest innovative considerations for future improvements of PCA pumps for elective surgical pediatric patients which is summarized in Table 1.

**Table 1:** Summary of safety concerns and potential solutions of patient-controlled analgesia.

**Concerns**	**Solutions**
Programming errors – Son et al. (6) – Rishoej et al. (8) – Campoe et al. (9)	– Smart pumps with barcode scanning – Computer provider entry – Establish reporting system – Regular audits
Programming protocol – Macintyre (10) – Craft (11)	– Standardization of protocol – Patient evaluation – Risk stratification – Staff education
Patient monitoring – Niesters et al. (12) – Langhan et al. (13) – Ronen et al. (14) – Craft (11) – Freemantle et al. (15) – Jay et al. (16)	– Assessment of sedation level – Smart pumps with oximetry and capnography
PCA by proxy – Howard et al. (17) – Craft (11) – Anghelescu et al. (18)	– Radiofrequency thumb tags – Patient and family education
PCA with continuous infusion – George et al. (19) – McNeely et al. (20) – Hayes et al. (21)	– Establish pediatric pain services

## The silent dangers of PCA use in children and adolescents

### Programming errors

PCA relies on the use of a program with different parameters such as bolus dose, lockout interval, hourly maximum dose and the drugs used, that will allow the patient to receive opioids in a predetermined pattern. Programming errors of the pump are therefore an important challenge concerning the safety of PCA (8). A recent retrospective study collecting the clinical records of 82,698 pediatric surgical patients demonstrated that 0.19% of cases experienced PCA device-related errors with the electronic programmable pump showing the highest error rate (6). The study also demonstrated that approximately 63% of cases with device-related errors experienced some type of adverse outcome such as nausea, vomiting, pruritus and inadequate analgesia. Although 0.19% may seem low, it represents 155 patients where errors could have been avoided and 96 patients whose preventable adverse events such as respiratory depression, may have been prevented (6). Most errors were associated with pump misprogramming which lead to serious unintended consequences such as oversedation, respiratory depression or undertreated pain. Since nurses oversee medication administration tasks and with the frequent distractions and interruptions occurring within the healthcare setting that increase the number of PCA medication errors, Campoe and Giuliano (9) investigated the impact of frequent interruption (2, 4, or 6 interruptions) on nurses' PCA programming performance. A mean error rate of 4.12% (10 errors) was observed, and although it may seem lower than the 20% of PCA medication errors reported in the United States of America (22), it reflects 10 patients who would have received incorrect medication dosing (9). Medication errors can pose a serious problem to the pediatric patient population because medication dosing is weight or surface area based (8). Although PCA device-related errors may not be frequent, they may prompt to serious consequences such as prolonged hospitalization and disabilities.

To address the issue of programming errors, dedicated smart pumps with barcode scanning of medications that sound and look alike may be useful during the initial set up and verification of the PCA program. Standard opioid concentration solutions should be used, and a verification system must be implemented to ensure that concentrations from the physician's order matches the nurse's programming and the pharmacy's stock. A medical information management system such as a computer provider order entry (CPOE) may enhance accuracy of medication orders and prevent programming errors (22). CPOE ensures that drug orders are accurate, performs drug allergy checks, and identifies drug interactions or wrong drug dosing (22). In an era of fast advances in technology and research on artificial intelligence, developing a system that could monitor a patient's demand or recognize their intention to treat their pain along with their vital signs and risks of adverse effects seems appropriate to look forward to. Future studies should evaluate the effectiveness of new technologies on PCA programming errors or adverse events in the clinical setting.

A reporting system needs to be established to know how many errors are made, what was the cause, and the frequency. This system would allow the pain services to reconsider their protocol and guidelines and may lead to a meeting with the staff to search for solutions to improve their quality of care. Regular audits should be implemented for statistical purposes to know the frequency of adverse events and medication errors related to PCA. This would allow the hospital to think critically for improvements in their establishment to minimize any future adverse events.

### Programming protocol

Another clinical challenge of PCA use is the lack of consensus on the optimal PCA parameter programming in the pediatric population (10). For example, the lockout interval of PCA is commonly set at 6 min based on the onset of action of most opioids being of 5 min. However, it is known that the peak effect of opioids varies between 7 and 15 min (11). Therefore, a 6-min lockout interval allows the patient to receive another dose of medication before the peak effect is reached. As a consequence, the patient is at risk for oversedation and serious adverse events such as respiratory depression.

Theoretically, a lockout interval of 10 min would allow for the full analgesic effect of opioids to take place before the next bolus dose is administered, decreasing overmedication and associated adverse events. However, previous studies have demonstrated that the influence of the lockout interval on pain relief and adverse effects had little to no effect (23, 24). Therefore, more studies regarding the influence of the lockout interval or the mechanism of action of opioids in PCA in children and adolescents are needed.

Preoperative self-reported measures that assess global psychological and behavioral profiles of the patients will identify those at risk of PCA misuse and adverse effects. This will allow for personalized PCA programming for patients in situations where they feel they have lost control such as after a surgery. In a case reported by Khan et al., psychiatric evaluation of an adolescent patient who received inadequate pain management revealed that the motivation behind increasing PCA doses was also to reduce anxiety. This behavior may complicate transition from PCA to oral analgesics and may cause the development of dependence during the arrest of medication (25). Online accessible validated tools assessing psychosocial parameters in the pediatric population are available and free of charge. Anxiety levels and pain catastrophizing, defined as exaggerating the pain experience than the general population, are among the best psychological predictors of patient's pain management after surgery (26, 27). Moreover, a preoperative psychophysical evaluation of the patient's somatosensory functioning with the use of quantitative sensory testing may provide insight into the postoperative pain experience of patients and their PCA dose-demand ratio (28). As an example, it was reported that the efficacy of patients' descending inhibitory pain system tested with a conditioned pain modulation paradigm before surgery predicted (*P* = 0.001) morphine consumption after surgery (29), suggesting a clinical value of such evaluation before surgery. A patient who demonstrates a tendency of catastrophizing a pain experience and is highly anxious, and whose descending inhibitory pain control is considered suboptimal would benefit of more preoperative education, monitoring, reassurance and most probably of the co-administration of an alpha 2-adrenergic receptor agonist that enhances the activity of descending inhibitory pain pathways (30). A limitation to patient evaluation is the reliability of one evaluation tool for all pediatric age ranges. There are accessible tools validated for specific age ranges (31). Therefore, it must be ensured that the appropriate method for patient evaluation is used and assessed in identifying the risk of PCA use and adverse effects in further studies.

A quality improvement project Cincinnati Children's Hospital Medical Center (2017) used risk stratification to stratify more than 90% of their patients for opioid misuse allowing earlier intervention in patients considered high risk (32). Risk stratification using a combination of subjective and semi-objective evaluations before surgery would pinpoint the patients who will properly participate in the management of their pain and those who need close monitoring and additional education on safe PCA use.

### Patient monitoring

Opioid-induced respiratory depression is the most serious complication of PCA therapy. Although the incidence is low (2.3%) (3), it may lead to respiratory arrest if not recognized and treated promptly (12, 33). Periodic spot checks of PCA patients remain the standard of care to detect opioid-induced respiratory depression and over-sedation. The issue with this method of monitoring is that patients are momentarily aroused by the patient-nurse interaction. Stimulation of the patient can transiently increase their respiratory rate and alertness and can result in an inaccurate reflection of their baseline clinical status. In this sense, an electronic safety net has been proposed for PCA patient monitoring with the use of both pulse oximetry and capnography. A study conducted by Langhan et al. analyzed the impact of capnography monitoring of 201 children in the post-anesthesia care unit. They observed that capnography monitoring leads to more effective staff interventions of respiratory depression (13). The Integrated Pulmonary Index (IPI) was recently developed and validated in a retrospective cohort of 523 patients in a variety of clinical settings (14). The IPI uses and integrates 4 parameters (end tidal CO_2_, respiratory rate, arterial oxygen saturation, and pulse rate) to improve respiratory monitoring. The IPI demonstrated good reliability in interpreting the respiratory status of patients in the validation cohort mainly consisting of adults and 1 pediatric cohort under procedural sedation (14). Unfortunately, the IPI has not been evaluated for use in the postoperative pediatric population. While capnography and the IPI are promising, these measures may not be the most effective to detect early opioid-induced respiratory depression, as hypoxia can occur in the absence of a low respiratory rate (11) and the overall result of a composite index may reflect certain individual components rather than the whole composite outcome (15). Therefore, other measures to improve patient monitoring must be considered including an increased surveillance of the patient and preoperative identification of risk factors for respiratory depression such as presence of respiratory problems, neurologic disease, renal disease, obesity, etc. (12, 16, 33).

The goal is early detection of opioid induced respiratory depression. Assessment of sedation level is the most reliable way to prevent opioid-induced respiratory depression as over-sedation usually precede respiratory depression (34). The Pasero Opioid Sedation Scale (POSS) has been validated in children (11, 35, 36). It is a simple and economic tool with lifesaving benefits that can be easily incorporated into periodic spot checks of PCA patients. Surveillance with a safety checklist and daily documentation of PCA opioid trends should be implemented.

Better development of pediatric monitoring such as integrating the overall response to analgesia with reported pain intensity is essential to increase safe PCA use. An innovative proposition would be a smart pump with pulse oximetry and capnography that decreases and/or stops opioid administration when parameters go beyond a predetermined limit and alarms healthcare staff.

### PCA by proxy

A major human error can occur during PCA by proxy, defined as the activation of the PCA by someone other than the patient, most commonly family members, caregivers, or friends. This must be differentiated from nurse-controlled analgesia (NCA) which is also defined as PCA delivered by proxy. NCA is suitable when PCA is contraindicated, such as the patient is unable to understand the concept of PCA or does not wish to control their analgesia. Although NCA is not as safe as PCA, nurses are better trained in postoperative pain management in comparison to a family member, caregiver or friend (17). PCA by proxy other than a nurse can be a highly dangerous practice as it negates a key safety measure of PCA use which is that a sleeping or sedated patient will not press the PCA button for more opioid administration (11). The individual pressing the PCA button for the patient may be a well-intentioned family member or caregiver intending to prevent pain upon awakening. Although PCA by proxy has been reported to lead to high patient satisfaction and compliance of the proxy in cases of children under the age of 5, children with neuromuscular impairment, or during painful procedures (18, 37), it may also lead to oversedation of the patient and other adverse events if the patient receives more bolus doses than needed. PCA dose limits over a certain time period are programmed, but there is no reliable method to predict the proper amount of opioids a patient needs for pain relief or for life-threatening events (10).

With technology within the healthcare setting advancing at a fast pace, it is expected to soon observe changes in the PCA devices. One could think of the idea that, based on the concept of unlocking a cell phone with a fingerprint, radiofrequency identification thumb tags of the patient on the PCA button would decrease the risk of oversedation and adverse events caused by a secondary individual administering the medication instead of the patient.

Education is a key element to improve safety for PCA by proxy. Staff and patient/parent education need to be emphasized. For patient/parent education, written and verbal preoperative education should be emphasized, because postoperative education or reinstruction is only ideal if the patient is alert and in a calm environment. Parents must understand that the PCA pump should only be used by the patient. Parents can get involved in their child's pain management, but they need to understand that even though their child may self-report high pain levels, if the pain is tolerable, then additional PCA doses are not warranted. Keeping a calm environment may reduce their anxiety and in turn, reduce the anxiety of the patient (38, 39). Warning signs should be placed at the head of the bed or attached to the pump stating that PCA can only be used by the patient. Evaluating adequate comprehension after PCA education should be implemented to assess the understanding of the patient and their parents.

### PCA with continuous infusion

PCA with background opioid infusion has been suggested as a solution for PCA by proxy, but studies on adults show that postoperative PCA with continuous infusion increased the risk of respiratory depression (19). Furthermore, limited data on continuous infusion in the pediatric population and low quality of studies has led to inconclusions for its advantage (19). A recent meta-analysis of randomized trials comparing PCA with and without background infusion showed that there was no significant difference in outcomes and background infusion did not pose an advantage over the use of PCA alone (20, 21). A simple but also important human error is mistaking the nurse bell for the PCA button. The patient will receive fewer bolus doses than allowed or needed, resulting in suboptimal pain control. Therefore, proper education of staff, caregivers and patients and signs at the bedside reinforcing the fact that the PCA button should only be activated by the patient is necessary.

Establishing pediatric pain services that are trained to recognize and assess, and safely and efficiently treat acute pain is ideal in every healthcare establishment. This pain service should be available 24 h per day, 7 days per week to ensure that any adverse events are treated in a timely response, and pain is managed thoroughly. The team should have a comprehensible treatment plan for each patient that is accessible to facilitate communication between the rest of the medical team (40).

## Conclusion

In summary, although PCA use has many advantages, concerns toward the safety of PCA devices remains. Careful patient selection and assessment, comprehensive education for patients, families and health care providers, PCA programming protocols with standardized solutions, and the use of appropriate monitoring are necessary for safe PCA use in the clinical setting. In addition, the practice should be regularly audited, and errors reported. As new technologies emerge, the use of smart pump technology and computer provider order entry will certainly decrease medication and programming errors.

## Author contributions

DO reviewed the literature and drafted the mini-review. AO and AT reviewed the literature, wrote parts of the review and contributed to the critical revisions. CF conceptualized the manuscript and contributed to the critical revisions and approved the final version.

### Conflict of interest statement

The authors declare that the research was conducted in the absence of any commercial or financial relationships that could be construed as a potential conflict of interest.
